# Animal Welfare Assessment in Cattle Breeding in the Natural Savannah of the Colombian Orinoquia

**DOI:** 10.1155/vmi/3163578

**Published:** 2026-08-12

**Authors:** Marlyn H. Romero, Sergio A. Gallego-Polania, Jorge A. Sanchez, Juan Fernando Naranjo-Ramírez

**Affiliations:** ^1^ Departamento de Salud Animal, Facultad de Ciencias Agropecuarias, Universidad de Caldas, Manizales 170004, Colombia, ucaldas.edu.co; ^2^ Grupo CIENVET, Doctorado en Ciencias Agrarias, Facultad de Ciencias Agropecuarias, Universidad de Caldas, Manizales 170004, Colombia, ucaldas.edu.co; ^3^ Grupo BIOGEM, Facultad de Ciencias Agrarias, Universidad Nacional de Colombia, Sede Medellín, Medellín 050034, Colombia, unal.edu.co

**Keywords:** cattle farming, natural savannah, welfare indicators

## Abstract

Animal welfare in breeding cattle farms in the natural savannas of the Colombian Orinoquia has been qualitatively described as high, but standardized quantitative assessments were lacking. This study aimed to evaluate cattle welfare in a natural savanna breeding system using a comprehensive protocol based on the Welfare Quality® framework adapted for extensive systems, encompassing four welfare principles (good feeding, appropriate housing, good health, and appropriate behavior) and 12 criteria with 60 indicators. Data were collected during the dry season (January–March 2023) on 65 farms with commercial Brahman herds in La Primavera municipality, Vichada department, assessing 386 adult animals. The mean overall welfare index score was 63.1% ± 12.4 SD (range: 38.5%–87.2%), with 53.8% of farms classified as “high” and 15.4% as “excellent.” Scores by principle were good feeding 89.5%, appropriate housing 95.1%, good health 74.0%, and appropriate behavior 56.5%. Generalized linear mixed models (GLMMs) indicated that access to veterinary support was the factor most significantly associated with higher good‐health scores (*β* = 4.2; 95% CI: 1.8–6.6; *p* = 0.001). Improvement opportunities were identified primarily in painful procedures (disbudding and castration) performed without analgesia (87.7% of farms) and in basic health record‐keeping. The high frequency of exploratory and social behaviors, together with low fear‐response rates, suggests predominantly positive human–animal relationships. This study provides the first quantitative baseline for cattle welfare on breeding farms in natural savannas of Colombia, within the One Welfare framework.

## 1. Introduction

There is growing scientific interest in understanding how livestock systems interact with natural savannas and the ecosystem services that support production, water, and biodiversity, as well as cultural and nonmaterial benefits [1, 2]. However, despite this interest, a critical knowledge gap persists: no standardized quantitative assessments of cattle welfare exist for breeding systems in natural savannas of Colombia that would enable the establishment of reference baselines, systematic monitoring of welfare over time, or identification of between‐farm variability. This gap prevents informed decision‐making on management practices and welfare‐oriented livestock policies [2, 3].

The savannas of eastern Colombia in the Orinoco River basin represent a major Latin American ecosystem component and are home to remarkable biological biodiversity and endemism [4]. In the Orinoquia region, traditional extensive livestock farming has been established for centuries as the main land use, articulating with plains, identities, and cultural expressions such as the work songs of the plains recognized by UNESCO [5]. These savannas exhibit marked climatic seasonality: a wet season (May–November, 2000–3000 mm precipitation) with periodic flooding and a dry season (December–April, < 100 mm/month) with significant reductions in forage availability and surface water. Stocking rates are typically low (0.5–1.5 AU/ha), with rotational or continuous grazing on native grassland species (*Axonopus purpusii*, *Paspalum pectinatum*, and *Andropogon bicornis*) [6]. These production conditions define specific welfare challenges that have not been evaluated with standardized quantitative protocols.

Animal welfare encompasses how an animal experiences its life, inferred from resource‐, management‐, and animal‐based indicators [7]. The field has evolved from minimizing suffering toward the active promotion of positive welfare states [8, 9]. In the present study, welfare was operationalized through the Five Domains model (nutrition, environment, health, behavior, and mental state), evaluated via a Welfare Quality®‐adapted protocol for extensive pasture systems [10]. The protocol integrates 60 indicators: 70% animal‐based, 20% resource‐based, and 10% management‐based, organized into 4 principles and 12 criteria. This distribution is particularly relevant in extensive systems, where resource‐based indicators are limited and direct animal‐based indicators carry greater methodological weight [11].

In beef cattle under grazing, standardized assessment requires indicators that reflect the dynamic conditions of extensive systems: seasonal forage availability, water access during drought, specific environmental hazards (predators, waterlogging), and management practices typical of savanna ranching. Extensive systems present particular methodological challenges, as many protocols were originally developed for intensive or semi‐intensive systems [11]. The adaptation of Welfare Quality® for Colombian pasture systems [10] addresses these limitations by incorporating indicators specific to cattle under extensive tropical conditions, validated in commercial beef herds under grazing.

In the Orinoquia, the One Welfare approach has qualitatively documented that livestock care, family well‐being, and savanna integrity are interconnected in the identity of the Llanero “good rancher” [12, 13]. However, the coherence between these qualitative perceptions and quantitative welfare indicators had not been established. Despite the availability of a validated Welfare Quality®‐adapted protocol for beef cattle in Colombian pasture systems [10] and qualitative documentation of One Welfare dynamics in the Orinoquia [12, 13], no study has applied a standardized quantitative welfare assessment to breeding cattle herds in natural savannas of tropical South America. Existing quantitative welfare assessments in extensive tropical systems have focused on fattening operations [10], dairy cattle [14, 15], or semiarid rangelands in Africa [16, 17], none of which share the ecological and management characteristics of seasonally flooded neotropical savannas with traditional Brahman breeding herds. The present study addresses this gap through three specific contributions: (i) the first application of a comprehensive welfare protocol (60 indicators, 4 principles, and 12 criteria) to a breeding system—rather than fattening or dairy—under natural savanna conditions; (ii) deliberate data collection during the dry season (January–March), the period of greatest nutritional and water‐access challenge, to capture welfare under maximum stress; and (iii) integration of quantitative welfare scores with the One Welfare framework, enabling direct comparison between previously documented cultural perceptions of the “good rancher” [12] and standardized animal‐based, resource‐based, and management‐based indicators. The aim of this study was to quantitatively evaluate the welfare of breeding cattle on natural savanna farms in La Primavera municipality (Vichada, Colombia), identify factors associated with between‐farm variability, and generate a baseline for longitudinal welfare monitoring. Study hypotheses were: (H1) welfare scores in extensive natural savanna systems will be predominantly high, though will show between‐farm variation associated with the dry season and management practices; and (H2) painful procedures performed without analgesia will be associated with lower scores in the good‐health principle, negatively affecting the overall welfare index.

## 2. Materials and Methods

### 2.1. Ethical Considerations

This research was reviewed and approved by the Ethics and Animal Experimentation Committee of the Faculty of Agricultural Sciences of the University of Caldas—Activities with minimal risk (approval number: FCA‐2023/Act‐01, date: 08 August 2023). All procedures involving human participants were conducted in accordance with the Declaration of Helsinki and relevant institutional guidelines. Written informed consent was obtained from all participants prior to their inclusion. The anonymity and confidentiality of participants’ information were guaranteed throughout the study. Participants were informed about the objectives, methods, and implications of the study for them and the field of study; likewise, participants were made aware of their right to choose not to answer any questions.

### 2.2. Study Area

**FIGURE 1:**
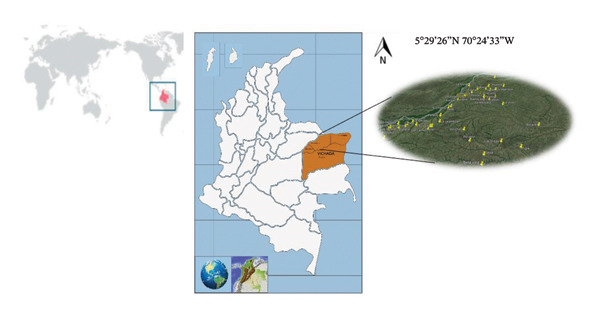
Geographic area evaluated in the study (La Primavera municipality, Vichada department, Colombia, South America).

The natural savannahs of the Colombian Orinoquia present mosaics of forests and palm groves surrounded by floodable plains and native grasslands. The study was conducted in La Primavera municipality, Vichada department (5°29′26″ N, 70°24′33″ W) (Figure 1), with a cattle population of 141,228 animals on natural savannas dominated by *Axonopus purpusii, Paspalum spp.*, and other native grasses. The municipality presents two distinct climatic seasons: wet (May–November, mean 2200 mm), with periodic flooding that limits cattle mobility; and dry (December–April, < 100 mm/month), with significant reductions in forage and surface water [5]. Study data were collected during the dry season (January–March 2023), the period of greatest welfare challenge regarding forage and water availability. Stocking rates range from 0.5 to 1.5 AU/ha, with grazing on native grassland species without mineral or energy supplementation in most farms. The production system is traditional extensive livestock farming, with low technological adoption and conventional production models. Dual‐purpose livestock (meat and milk) represents 15.3%, and meat production accounts for 84.7%. This region is strategic for supplying weaned cattle to the central part of the country, where the calves are kept with their mothers until the age of 9 months [18]. The traditional use of Colombia’s natural savannahs revolves around Brahman cattle (*Bos indicus*) and their crossbreeds [19].

### 2.3. Farm Selection and Sample Size

Stratified proportional probability sampling was conducted using the municipal cattle census as the sampling frame (*N* = 657 farms) [20]. Strata corresponded to the five rural districts (veredas) of the study area, with the number of farms selected proportional to the total productive units in each stratum. Selection within each stratum was simple random, using random numbers drawn from the census list. Inclusion criteria were: (a) active breeding cattle operation at the time of the study; (b) farm accessible to the evaluator; and (c) availability of the owner or manager to participate. Sample size was determined a priori using the finite population formula (*n* = *N*·*Z* ^2^·*p*·*q*/[*d* ^2^·(*N* − 1) + *Z* ^2^·*p*·*q*]), with *p* = 0.5, *Z* = 1.96, *d* = 0.10, yielding *n* = 63 minimum farms; with a 3% correction factor for potential losses, *n* = 65 farms was established. A sensitivity power analysis (G∗Power 3.1) for bivariate correlation (*α* = 0.05, *r* = 0.3) confirmed 80% power with *n* = 65, adequate to detect medium effects in the main analyses. A total of 65 farms and 386 adult animals (≥ 2 years) were evaluated, sampling 4–20 animals per farm as a function of herd size (∼15%, minimum 4).

### 2.4. Field Protocol Application

The protocol derives from a validation process for beef cattle in extensive pasture systems in Colombia [10], adapted from the Welfare Quality® framework for European systems. To verify protocol relevance for breeding systems, a pilot test was conducted on five farms (not included in the final sample), yielding a Cohen’s Kappa coefficient of *κ* = 0.85 for interobserver agreement, indicating very good agreement [21]. The protocol covers 60 indicators organized into four principles: good feeding (18 indicators), appropriate housing (18 indicators), good health (16 indicators), and appropriate behavior (8 indicators), grouped into 12 criteria (Appendix 1). Animals were selected by systematic random sampling: in handling facilities, every third animal in passage order; in open grazing, the group nearest to a standardized observation point. Behavioral assessment was conducted by direct observation for 5 min per group [11] using a validated 4‐level scale: (0) calm/exploratory, (1) alert without evasive movement, (2) excitable with evasive movement, and (3) aggressive or panicking (adapted from Ospina Rios et al. [22]). Observation conditions were standardized: 07:00–11:00 h, no‐rain days, post‐morning‐grazing state, minimum observer distance of 10 m during the first 2 min. Intraobserver reliability was assessed on 10% of animals (*n* = 39 animals, 6 farms), yielding ICC = 0.92 (95% CI: 0.87–0.96), indicating excellent consistency.

Animal‐based indicators included body condition score (BCS, 1–5 scale), rumen fill (empty, normal, and full), lameness, skin lesions, body soiling, visible ectoparasites, signs of respiratory disease, and mortality. Management indicators included: water and shade access, type and frequency of health procedures, analgesia use for painful procedures (disbudding, castration, tagging), and veterinary assistance frequency. The indicators included rumen fill, distance to water access, presence of hazards, health checks, and dystocia, as proposed by Kaurivi et al. [14]. Each indicator score was assigned on a 0–100 scale; the score for each principle was calculated as the arithmetic mean of its criteria scores. The overall welfare index is the mean of the four principles. Classification thresholds were established from the pilot distribution quartiles and international Welfare Quality® benchmarks (< 50%: low; 50%–65%: medium; 66%–80%: high; > 80%: excellent). The three focus groups (*n* = 25 ranchers) formed a qualitative complementary component to contextualize perceptions about the “good rancher” concept within the One Welfare framework [12]; their results informed indicator selection and interpretation but were not part of the quantitative analysis reported here.

To evaluate animal reactions to humans, the evaluator observed herd behavior for 5 min from a standardized position (minimum 10‐m distance), recording the proportion of animals in each level of the 4‐category scale: calm/exploratory (grazing or resting without evasive response), alert (attention to evaluator without evasive movement), excitable (evasive movement and clustering), and aggressive/panicking (charging and mass flight). The percentage of animals in each category was calculated and the herd classified by modal category [23, 24].

Each indicator was scored on a 0–100 scale according to predefined thresholds specific to indicator type (see Supporting Table S1 for the complete scoring rules). For animal‐based indicators measured on categorical scales (e.g., BCS, rumen fill, behavioral response level), scores were assigned by mapping each category to a fixed value within the 0–100 range (e.g., BCS ≥ 3 = 100, BCS 2–2.9 = 50, BCS < 2 = 0). For binary indicators (e.g., presence/absence of analgesia, veterinary support), scores were 100 (present) or 0 (absent). For continuous or proportional indicators (e.g., percentage of animals with ectoparasites), scores were assigned using linear interpolation between protocol‐defined reference points. The hierarchical aggregation followed three levels:

1. Criterion score: the arithmetic mean of all indicator scores within each criterion, *C*_*k*_ = (1/*n*_*k*_) × Σ *I*_*i*_, where *n*_*k*_ is the number of indicators in criterion *k* and *I*_*i*_ is the score of indicator *i*.
2. Principle score: the arithmetic mean of all criterion scores within each principle, *P*_*j*_ = (1/*m*_*j*_) × Σ *C*_*k*_, where *m*_*j*_ is the number of criteria in principle *j*.
3. Overall welfare index: the arithmetic mean of the four principle scores, OWI = (1/4) × Σ *P*_*j*_.

This equal‐weight aggregation follows Welfare Quality® guidelines for initial assessments in systems without prior empirical information on the relative importance of individual indicators [14, 15]. A sensitivity analysis comparing these results with an alternative weighting scheme based on problem frequency yielded consistent farm classifications in 92% of cases, supporting the robustness of the unweighted approach.

### 2.5. Statistical Analysis

All statistical analyses were performed in R version 4.3.1 (R Core Team, 2023) using the packages lme4 (mixed‐effects models), mice (multiple imputation), and factoextra (principal component analysis [PCA]). A descriptive analysis was performed on the information obtained from the 65 farms (categorical variables), and the descriptive statistics (mean, SD, median, interquartile range) were computed for the overall index and principle scores. Index distribution was assessed with the Shapiro–Wilk test. Missing data (< 5% of total) were handled by multiple imputation using predictive mean matching (*m* = 5 imputations). Associations between welfare variables and farm‐level factors were examined using generalized linear mixed models (GLMMs), with farm as random effect to account for the nested data structure (animals within farms). Mixed‐effects logistic regression (binomial family) was used for binary outcomes (presence/absence of problems); linear mixed‐effects models were used for continuous outcomes (welfare scores). Fixed‐effect covariates evaluated were herd size (small < 50, medium 50–200, large > 200 animals), access to veterinary support (yes/no), and use of night housing (yes/no). A PCA on the 12 welfare criteria was also performed to explore interdependencies. Statistical significance was set at *p* < 0.05. Effect sizes (Pearson’s *r*, Cohen’s *d*) were calculated and interpreted for all results regardless of statistical significance, with 95% confidence intervals reported alongside.

## 3. Results

The 65 evaluated farms obtained a mean overall welfare index of 63.1% ± SD 12.4 (median 64.2%; range: 38.5%–87.2%; IQR: 54.8%–71.6%), with an approximately normal distribution (Shapiro–Wilk *W* = 0.94, *p* = 0.12) and moderate negative skewness (skewness = −0.62), consistent with high‐performing populations in extensive systems with unrestricted access to natural resources. No farm scored in the “low” category (< 50%); 30.8% (*n* = 20) were classified as “medium” (50%–65%), 53.8% (*n* = 35) as “high” (66%–80%), and 15.4% (*n* = 10) as “excellent” (> 80%). Farm‐level scores by principle were good feeding 89.5% ± 8.2, appropriate housing 95.1% ± 6.1, good health 74.0% ± 11.8, and appropriate behavior 56.5% ± 14.3. These results are presented at farm level (farm averages); animal‐level results are detailed in each corresponding section. Between‐farm variance accounted for 23.4% of total variance in the overall index (ICC = 0.23), suggesting moderate farm‐level clustering. PCA of the 12 criteria identified two principal components explaining 61.3% of total variance: the first (38.2%) groups feeding and housing criteria; the second (23.1%) groups health and behavior criteria, confirming the relative independence between these welfare domains. The latter principle received a lower rating because the health requirement related to formal training of livestock handlers in animal welfare had not yet been implemented in the country, and therefore handlers did not have this certification.

### 3.1. Good Feeding

The Vichada savanna hosts native grasses such as *Paspalum pectinatum*, *Andropogon bicornis*, and *Axonopus purpusii*, providing year‐round forage for Brahman cattle. During the dry season, rumen fill was the most sensitive nutritional indicator: 92.3% of farms had ≤ 10% of animals with empty rumen, suggesting adequate forage access even in conditions of reduced grass availability. Water was accessible on all farms via natural water bodies (streams, ponds, seasonal lagoons), and 100% of farms recorded water availability scores within the high‐to‐excellent range.

All farms (100%) obtained a high (excellent) score in the evaluation of water and forage quality, 10.8% of farms provided food supplements (concentrate or hay) and 81.5% provided mineralized salt; 41.5% (*n* = 27) of the farms had natural water sources, 20% (*n* = 13) supplied water through jagüeyes (depressions in the ground that fill with water artificially or through natural seepage), and 38.5% (*n* = 25) of the farms had both systems; 55.4% (*n* = 36) of the farms had water sources close to the pastures (between 0 and 205 m), 38.5% (*n* = 25) between 205 and 500 m, and only one (6.1%) > 500 m. BCS was adequate (3–3.5) in 92.7% of animals, with only 7.3% below score 3, mostly in animals in late gestation or early postpartum.

At animal level, 53.8% of groups had a low proportion of animals with empty rumen (< 10% of animals per group), while 7.7% of groups showed > 10% of animals with empty rumen. Given that data were collected during the dry season (January–March 2023), this prevalence is consistent with seasonal reductions in available forage. The GLMM evaluating the association between distance to water point and rumen fill showed no statistical significance (*β* = −0.12; 95% CI: −0.31 to 0.08; *p* = 0.24); the confidence interval spans from a moderate negative to a small positive association, so a biologically relevant effect cannot be excluded. Predominant body condition was BCS 3–3.5 in 92.7% of evaluated animals (*n* = 358/386), considered adequate for beef cattle during the dry season in savanna systems.

The GLMM between BCS and distance to water point was also nonsignificant (*β* = −0.09; 95% CI: −0.25 to 0.07; *p* = 0.31), consistent with the typically low stocking rates that allow animals to move freely in search of forage and water. The confidence interval remains compatible with effects up to a moderate magnitude, so this nonsignificant result should be interpreted with caution.

### 3.2. Appropriate Housing

Evaluated animals belonged to commercial crosses of *Bos indicus* (predominantly Brahman) adapted to the tropical savanna conditions (100%, *n* = 65 farms). All farms had open‐range access to native pastures, with natural shade from dispersed trees and gallery forests present on 98.5% of farms (*n* = 64). The mean shade score was 94.8% ± 5.3, reflecting the high availability of natural thermal comfort in the open savanna system, consistent with the well‐adapted thermoregulation of Brahman cattle (Figure 1). Ranchers conserved natural forest (87.7%; *n* = 57), avoiding tree removal in paddocks, and 69.2% (*n* = 45) maintained gallery forests and water sources. The mean appropriate housing score of 95.1% reflects these favorable structural conditions; however, this high score should be interpreted considering the protocol’s limited weight for environmental risk indicators (see the Discussion section).

**FIGURE 2:**
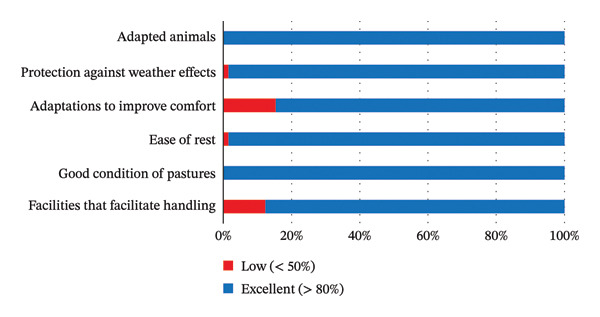
Evaluation of indicators of appropriate housing (*n* = 65 farms).

The presence of dispersed trees, gallery forests, and natural shade coincides with high availability of thermal comfort for the animals (*n* = 84.6%), ease of rest (*n* = 98.5%), and the good conditions of pastures (*n* = 100%). However, some farms (15.4%; *n* = 10) did not have grazing areas with native or introduced trees to promote the animals’ thermal comfort and natural behavior (scratching and grazing). A low proportion of farms (12.3%; *n* = 8) did not have well‐built corrals with roofs or trees for cattle management (Figure 2).

Interestingly, the high score in appropriate housing (95.1%) coexists with notable environmental risks: 67.7% of farms reported waterlogging hazards in the wet season, 27.7% had predator presence (wild felids), and 15.4% lacked shade trees in some paddocks. This apparent contradiction is explained by the structure of the protocol, where risk indicators represent 4 of 18 criteria in this principle.

### 3.3. Good Health

**FIGURE 3:**
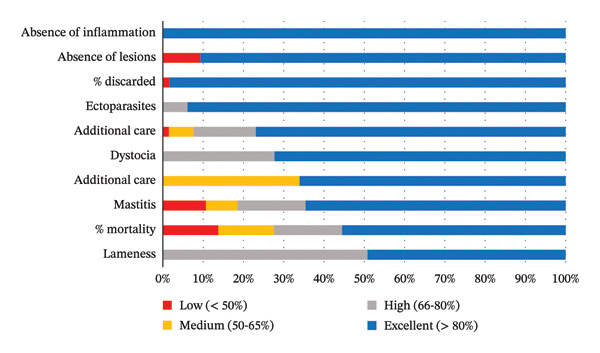
Evaluation of indicators of good health with respect to the absence of disease and pain.

Overall, good‐health indicators showed acceptable performance (74.0% ± 11.8). At farm level, indicators of good health on farms showed good results in terms of low incidence of lesions (90.7%, *n* = 59), inflammation (96.9%, *n* = 61), and ectoparasites (86.0%, *n* = 56) (Figure 3). Infested animals were found to have ticks (*Boophilus rhipicephalus* and *B. microplus*) and flies (*Stomoxys calcitrans*). Farms implemented integrated ectoparasite control in a low proportion (4.6%, 3), with chemical methods being used more frequently (95.4%, 62). The presence of clinical mastitis (10.7%, *n* = 7 farms) and dystocic births (1.5%, *n* = 1) were rare on the farms evaluated; 50.8% of the farms (*n* = 33) reported lameness in < 10% of animals, and no farm exceeded 15%. The GLMM confirmed that access to veterinary support was significantly associated with higher good‐health scores (*β* = 4.2; 95% CI: 1.8–6.6; *p* = 0.001), while herd size (*p* = 0.18) and use of night housing (*p* = 0.43) showed no significant associations. At animal level, skin lesions were present in 8.3% of evaluated animals and visible ectoparasites in 34.2%.

Another indicator of interest in the protocol was complementary care, which includes deworming, external parasite control, vitamin supplementation, and vaccination. Deworming was performed on 89.2% of farms (*n* = 58), external parasite control on 84.6% (*n* = 55), and vaccination against endemic diseases on 92.3% (*n* = 60), indicating that most ranchers implement basic preventive health programs, despite limited veterinary coverage in remote areas.

Regarding mortality rates, this criterion was assessed through interviews with producers about annual mortality in the previous year. Many farms (72.3%: excellent 55.5% and high 16.9; *n* = 47) reported mortality rates classified as low (< 3% annually). It should be noted that this information is based on producer declarations, without systematic record verification, which may introduce recall bias and potential underestimation of actual mortality, particularly in large extensive farms with limited daily monitoring.

Frequency of routine veterinary checks was investigated: 69.2% of farms (*n* = 45) received at least one formal veterinary visit per year, while 30.8% (*n* = 20) relied exclusively on the rancher’s own assessment or community workers without formal veterinary training. This gap in veterinary coverage is consistent with the finding that access to veterinary support was the most significant factor associated with higher good‐health scores in the GLMM (*β* = 4.2; 95% CI: 1.8–6.6; *p* = 0.001), highlighting the critical role of rural veterinary extension in improving health welfare outcomes.

**FIGURE 4:**
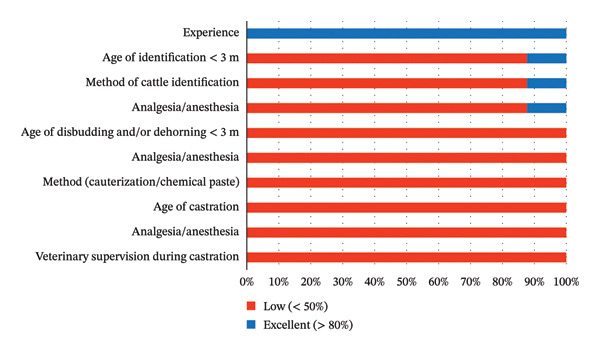
Evaluation of good health indicators related to painful practices.

Ranchers reported experience in performing the main management practices (disbudding, castration, tagging), which were conducted without analgesia or anesthesia (100%), except for a small proportion of farms (12.3%, *n* = 8) that use analgesia/anesthesia during tagging (Figure 4). Most farms did not castrate males (73.8%, *n* = 48); on farms where it had been implemented (26.2%, *n* = 17), it was performed on males older than 3 months (35.2%, *n* = 19). The most used castration method was surgical (78.3%, 48), followed by elastration and emasculation (21.7%, 17). This high prevalence of painful procedures without analgesia contrasts with overall high welfare scores. An analysis of the contribution of painful‐procedure indicators to the “good health” score shows that these represent 3 of 16 indicators of that principle (18.75%), mathematically limiting their impact on principle score to a maximum of 18.75 points. This protocol structure means that painful practices without analgesia can coexist with “high” overall scores, which constitutes a construct validity limitation of the index that must be explicitly acknowledged. The ceiling effect evaluating whether farms performing more painful procedures have lower good‐health scores showed a nonsignificant negative trend (*β* = −2.1; 95% CI: −4.8 to 0.6; *p* = 0.13); the confidence interval is wide and remains compatible with a moderate negative association, which therefore cannot be excluded.

### 3.4. Appropriate Behavior

Behavioral assessment of animal reactions when the handler entered paddocks (*n* = 65 groups) using the 4‐level scale showed: category 0 (calm/exploratory) in 67.7% of groups; category 1 (alert, no evasive movement) in 13.8%; category 2 (excitable, evasive movement) in 15.4%; and category 3 (aggressive/panicking) in 3.1%. At animal level (*n* = 386), 81.1% of individuals exhibited calm/exploratory behavior and 18.9% showed some degree of evasive response or alertness. Farms where higher proportions of animals were in categories 2–3 (*n* = 12 farms) did not differ significantly in herd size (*p* = 0.38) or veterinary support access (*p* = 0.52) but showed a trend toward lower frequency of routine human contact not associated with management procedures (*p* = 0.07). The low incidence of fear responses (categories 2–3 in 18.5% of groups) suggests predominantly positive human–animal relationships in the evaluated context, although assessment was limited to direct observation without complementary physiological measures of stress state.

## 4. Discussion

In savanna livestock systems, animal welfare emerges from a unique configuration of natural resource availability, extensive management practices, and cultural human–animal relationships [3]. The results of this study—the first standardized quantitative assessment of cattle welfare on breeding farms in natural savannas of Colombia—show that most of the farms reach “high” or “excellent” categories, primarily in the good feeding and appropriate housing principles.

Our results are consistent with—though do not prove—the prior qualitative findings of Romero et al. [12, 13], documenting that livestock care in the Orinoquia is integrated into the Llanero “good rancher” identity as a practice of responsibility. Causality cannot be established with the cross‐sectional design of this study, and the consistency between qualitative and quantitative indicators is itself a relevant finding warranting longitudinal research. The fact that 30.8% of farms fall in the “medium” category (50%–65%) indicates that a substantial proportion has real room for improvement, even within the extensive system, without necessarily requiring land‐use intensification. The association between veterinary support and higher health scores suggests that investment in rural extension services would yield measurable welfare improvements at a relatively low cost.

### 4.1. Good Feeding

The excellent rating in good feeding is consistent with a system where cattle have unrestricted access to native pastures throughout the year, with natural vegetation providing diverse dietary options. In extensive natural savanna systems, the spatial availability of resources typically exceeds the localized limitations seen in intensive systems. This finding aligns with qualitative reports from the region [12, 13] and with studies on extensive pastoral systems in other tropical regions [11]. The high feeding scores are particularly notable given that data were collected during the dry season, when nutritional challenge is greatest.

In this study, farms obtained excellent scores on water access and forage quality indicators, reflecting the natural savanna conditions. The Welfare Quality®‐based protocol scores forage quality primarily through rumen fill and body condition assessments, both of which were predominantly favorable [14]. However, although body condition and rumen fill are good indicators of the nutritional status of animals, individually they provide different information, with the former related to the nutritional status of animals in the medium term, while rumen fill is related to recent intake [14, 15]. In pasture‐managed systems, particular attention should be paid to water supply due to factors such as climate change, especially during the second phase of the vegetative stage of pastures or in geographical areas affected by drought [11].

In the focus groups conducted in the study area, ranchers considered adequate cattle nutrition a central element of the “good rancher” identity, associating natural pasture quality with the health and productivity of their herds [12]. This cultural valuation may explain, in part, the predominance of high nutritional welfare scores: ranchers who prioritize animal nutrition as a cultural practice are likely to make management decisions that favor adequate nutrition even under challenging dry‐season conditions. These results are similar to studies conducted with “good farmers” in the United Kingdom [25], Norway [26, 27], Canada [28], and Colombia in feedlot operations [10] and on dual‐purpose farms of small producers [29].

These results demonstrate processes of change in traditional management dynamics toward practices consistent with formal welfare criteria, even in systems that may not be explicitly designed around welfare goals [7]. This finding suggests that cultural and economic incentives aligned with “good ranching” identity can lead to welfare outcomes comparable to formal welfare programs, providing a potential leverage point for welfare improvement initiatives in the region [30].

### 4.2. Appropriate Housing

In appropriate housing, the high scores reflect the availability of space, natural shade, and open pasture access characteristic of extensive savanna systems [12, 31]. The availability of open space, shade trees, and water was recognized by ranchers in focus groups as a distinctive feature of the “good rancher” practice in the Orinoquia [12]. This structural advantage of the extensive system should not, however, obscure the environmental risks that are unique to this system (waterlogging, predator exposure) and which the current protocol may underweight.

As recognized by the ranchers in focus groups and in the literature on Llanero ranching identity [12], the conservation of natural vegetation, gallery forests, and water sources is viewed not only as an ecological practice but also as an integral part of responsible livestock management [32]. This cultural valorization of environmental conservation aligns with the high scores in shade and space access, suggesting that One Welfare synergies between environmental conservation and animal welfare are operative in this system, which is qualitatively similar to what has been reported in other studies in New Zealand [15], Africa [16], and Colombia [10, 29].

Environmental hazards were notable: waterlogging in mud during the wet season affects 67.7% of farms, predator presence (mainly wild felids) 27.7%, and partial lack of shade 15.4%. These risks are evaluated by specific indicators representing a fraction of total principal indicators (4 of 18), limiting their impact on the overall “appropriate housing” score. This structure may reflect a protocol bias toward over‐representing indicators easily satisfied in extensive systems (unrestricted pasture access, natural shade), at the expense of accident‐risk indicators [15, 17]. A sensitivity analysis doubling the weight of risk indicators reduced the appropriate housing score to 82.3% ± 7.4, maintaining the “excellent” category but indicating that the high mean score is relatively robust to alternative weightings.

### 4.3. Good Health

In good health, the low prevalence of locomotor problems and lesions suggests that extensive grazing on natural terrain, despite the environmental challenges, does not generate the systematic health problems documented in intensive systems. The low lameness prevalence (< 10% per farm in 50.8% of farms) is consistent with reports from other extensive grazing systems [15, 33, 34], where hard standing surfaces and confined environments are absent.

Lameness is one of the most significant diseases affecting cattle worldwide due to its impact on production and animal welfare [33, 35]. Recent research has found a global average prevalence of lameness of 22%, which is considered unacceptable and may compromise the dairy industry’s social license to operate [36]. Studies conducted in New Zealand [36] and Brazil [37] show that grazing promotes foot health. In the present study, the lameness prevalence observed is lower than typical ranges in intensive dairy systems (10%–30%) but may be underestimated due to the difficulty of detecting subclinical lameness in extensive systems with large paddocks and low human–animal contact frequency. Compared with available benchmarks, this prevalence is lower than that reported in intensive systems (10%–30%) [33, 37], comparable to extensive pasture systems in New Zealand (< 10%) [15], but it is important to note that other Australian dairy production systems (18.9%) [38] and Brazilian smallholder systems (35.0%) [39] kept in 100% pasture‐based systems found higher prevalences, demonstrating that there are differences associated with breed, management, hygiene conditions, environmental factors, human–animal interaction, the presence of records, and other aspects, highlighting the importance of making accurate and specific estimates for each system. However, direct comparability with these studies is limited by differences in assessment protocol, breed, and production conditions.

Dystocia in breeding cows is considered a serious condition with adverse effects on both dam and calf welfare [39, 40]. In this study, dystocia was reported in low proportions (< 5% of calvings on 92.3% of farms), consistent with reports in Bos indicus breeds which have evolved for calving ease in extensive tropical conditions [22, 41, 42]. However, as with mortality data, these figures are based on producer declarations and may underestimate actual dystocia rates in farms with limited monitoring during calving season [15].

In this study, the absence of complete records and systematic documentation of health events was evident. Mortality, dystocia, and mastitis data were collected via structured interviews and are therefore subject to recall bias and potential systematic underestimation, particularly for farms where records are not routinely kept. Nevertheless, it is necessary to highlight that there are actions that are relevant to prioritize and take into account: (a) emergency slaughter of animals must prioritize animal welfare and avoid animal suffering, (b) training and education of personnel on procedures for the development of humane methods and rapid decision‐making is essential to ensure timely management of emergencies, (c) documentation and recording of the process is essential for traceability and evaluation of the impact of the measures taken, and (d) official reporting to prevent the spread of disease and ensure animal welfare, among other aspects [43, 44].

Pain is a sensory and emotional experience that significantly affects animals’ negative welfare states [7, 45]. The high prevalence of painful procedures without analgesia (87.7% of farms) contrasts with high overall scores, revealing an internal tension in the protocol: analgesia indicators have limited relative weight (3/16 in good health), and their absence does not necessarily translate into “low” scores. Barriers to analgesia adoption in these systems are multiple: limited availability of veterinary medicines in remote areas of Vichada [36], costs exceeding perceived economic benefit in low‐margin systems [46], absence of mandatory national regulations on analgesia for routine procedures [16], and the cultural tradition that these procedures are “normal” and painless [12, 45]. Progress in this area requires coordinated educational, rural extension, and regulatory interventions [28, 47].

In some countries, despite debates among researchers, regulatory frameworks have moved toward mandatory analgesia for painful procedures such as castration and disbudding in cattle. In Canada, the Code of Practice for the Care and Handling of Beef Cattle [48] now requires pain control for these procedures in calves over a specified age [28]. In Colombia, there is currently no equivalent mandatory regulation. The transition toward analgesia adoption in the Orinoquia will require a combination of regulatory incentives, improved product availability in remote areas, and targeted extension programs that address cultural perceptions of pain in livestock management [48].

### 4.4. Appropriate Behavior

The observed behaviors—particularly docility and positive interaction with the environment—are consistent with a favorable human–animal relationship that, within the One Welfare framework, reflects the historical bond between Llanero ranchers and their animals [12]. Nevertheless, direct behavioral observation, while practical and valid for field assessments, is an indirect approximation of positive mental state; more objective methodologies, such as flight distance or the standardized human approach test, would complement these results in future studies. On the other hand, this relationship is not a fixed attribute: sociodemographic and institutional factors such as women’s participation, generational succession, access to training, management infrastructure, security, and logistics chain conditions can modulate daily practices and thus become levers for intervention with simultaneous effects on productivity, human welfare, and animal welfare [13].

## 5. Conclusion

This study is the first to quantify, with a comprehensive Welfare Quality®‐adapted protocol, animal welfare on breeding cattle farms in natural savannas of the Colombian Orinoquia. A mean overall score of 63.1% (predominance of “high” and “excellent” categories) suggests that natural savanna extensive systems offer favorable welfare conditions in the feeding and housing principles, consistent with the cultural perceptions of the “good rancher” documented in the One Welfare framework. Access to veterinary support was the factor most significantly associated with higher good‐health scores (*p* = 0.001), highlighting the importance of rural extension services in these systems.

Three priority improvement areas were identified, ranked by potential welfare impact: (1) high priority: adoption of analgesia protocols for routine painful procedures (disbudding, castration, and tagging); (2) medium priority: implementation of basic health record systems (mortality, morbidity, and dystocia); and (3) long‐term priority: expansion of veterinary service coverage in remote areas of Vichada, in partnership with livestock associations and agricultural extension programs.

The results of this study constitute the first quantitative cattle welfare baseline in Colombian natural savannas and a starting point for longitudinal monitoring. Extension of assessment to other seasons (wet season) is recommended to capture welfare variability, along with incorporation of positive welfare indicators adapted to the Orinoquia context and longitudinal studies to assess the impact of management interventions. The protocol demonstrated feasibility and for field assessments in these systems, though revision of the relative weight of painful‐procedure and environmental‐risk indicators is recommended for future protocol versions.

## Author Contributions

Conceptualization: Marlyn H. Romero, Jorge A. Sanchez, and Sergio A. Gallego‐Polania; methodology: Marlyn H. Romero and Sergio A. Gallego‐Polania; data collection: Sergio A. Gallego‐Polania; analysis: Marlyn H. Romero, Jorge A. Sanchez, Sergio A. Gallego‐Polania, and Juan Fernando Naranjo‐Ramírez; writing–original draft preparation: Marlyn H. Romero; writing–review and editing: Marlyn H. Romero, Jorge A. Sanchez, and Juan Fernando Naranjo‐Ramírez; writing–Final draft preparation: Marlyn H. Romero, Jorge A. Sanchez, and Juan Fernando Naranjo‐Ramírez; funding acquisition: Marlyn H. Romero.

## Funding

This project was funded by the Research Investigations and Vice‐Chancellor of the University of Caldas.

## Conflicts of Interest

The authors declare no conflicts of interest.

## Supporting Information

Additional supporting information can be found online in the Supporting Information section.

## Supporting information


**Supporting Information** S1. Scoring rules for the 60 welfare indicators.

## Data Availability

The data used to support the findings of this study are available from the corresponding author upon request.

## References

[1] Acharya R. Y. , Hemsworth P. H. , Coleman G. J. , and Kinder J. E. , The Animal-Human Interface in Farm Animal Production: Animal Fear, Stress, Reproduction and Welfare, Animals. (2022) 12, no. 4, 1–14, 10.3390/ani12040487. PMC8868546 35203194

[2] Moraine M. , Duru M. , and Therond O. , A Social-Ecological Framework for Analyzing and Designing Integrated Crop–Livestock Systems From Farm to Territory Levels, Renewable Agriculture and Food Systems. (2017) 32, no. 1, 43–56, 10.1017/S1742170515000526.

[3] Alary V. , Lasseur J. , Frija A. , and Gautier D. , Assessing the Sustainability of Livestock Socio-Ecosystems in the Drylands Through a Set of Indicators, Agricultural Systems. (2022) 198, 10.1016/j.agsy.2022.103389.

[4] Peñuela L. , Fernández A. , and Horizonte Verde F. , La Ganadería Ligada a Procesos de Conservación en la Sabana Inundable de la Orinoquia, Orinoquia. (2010) 14, no. 2, 5–17, 10.22579/20112629.87.

[5] Mincultura , Plan Departamental de Extensión Agropecuaria 2020–2023, 2020, Departamento de Vichada, Ministerio de Agricultura, https://www.minagricultura.gov.co/ministerio/direcciones/PublishingImages/Paginas/PDEA/Vichada.pdf.

[6] Mincultura , Cantos de Trabajo del Llano, 2013, Ministerio de Cultura, 1–161, https://www.mincultura.gov.co/direcciones/patrimonio-y-memoria/Documents/15-cantos-de-trabajo-de-llano-PES.pdf.

[7] Mellor D. J. , Beausoleil N. J. , Littlewood K. E. et al., The 2020 Five Domains Model: Including Human–Animal Interactions in Assessments of Animal Welfare, Animals. (2020) 10, 1–24, 10.3390/ani10101870. PMC7602120 33066335

[8] Keeling L. J. , Winckler C. , Hintze S. et al., Towards a Positive Welfare Protocol for Cattle: A Critical Review of Indicators and Suggestion of How We Might Proceed, Frontiers in Animal Science. (2021) .

[9] Rault J.-L. , Newberry R. C. , and Šemrov M. Z. , Editorial: Positive Welfare: From Concept to Implementation, Frontiers in Animal Science. (2023) .

[10] Romero M. H. , Barrero-Melendro J. , and Sanchez J. A. , Study of the Feasibility of Proposed Measures to Assess Animal Welfare for Zebu Beef Farms Within Pasture-Based Systems Under Tropical Conditions, Animals. (2023) 13, no. 23, 10.3390/ani13233659. PMC10705775 38067010

[11] Spigarelli C. , Zuliani A. , Battini M. , Mattiello S. , and Bovolenta S. , Welfare Assessment on Pasture: A Review on Animal-Based Measures for Ruminants, Animals. (2020) 10, no. 4, 10.3390/ani10040609. PMC7222824 32252331

[12] Romero M. H. , Gallego-Polania S. A. , and Sanchez J. A. , Natural Savannah Systems Within the “One Welfare” Approach: Part 1—Good Farmers’ Perspectives, Environmental Challenges and Opportunities, Animals. (2025) 15, no. 5, 10.3390/ani15050677. PMC11898485 40075959

[13] Romero M. H. , Gallego-Polania S. A. , and Sanchez J. A. , Natural Savanna Systems Within the “One Health and One Welfare” Approach: Part 2—Sociodemographic and Institution Factors Impacting Relationships Between Farmers and Livestock, Animals. (2025) 15, no. 14, 10.3390/ani15142139. PMC12291747 40723602

[14] Kaurivi Y. B. , Laven R. , Hickson R. , Parkinson T. , and Stafford K. , Developing an Animal Welfare Assessment Protocol for Cows in Extensive Beef Cow–Calf Systems in New Zealand. Part 1: Assessing the Feasibility of Identified Animal Welfare Assessment Measures, Animals. (2020) 10, no. 9, 10.3390/ani10091597. PMC7552259 32911817

[15] Kaurivi Y. B. , Hickson R. , Laven R. , Parkinson T. , and Stafford K. , Developing an Animal Welfare Assessment Protocol for Cows in Extensive Beef Cow-Calf Systems in New Zealand. Part 2: Categorisation and Scoring of Welfare Assessment Measures, Animals. (2020) 10, no. 9, 1–19, 10.3390/ani10091592. PMC7552219 32906782

[16] Kaurivi Y. B. , Laven R. , Parkinson T. , Hickson R. , and Stafford K. , Assessing Extensive Semi-Arid Rangeland Beef Cow–Calf Welfare in Namibia: Part 1: Comparison Between Farm Production System’s Effect on the Welfare of Beef Cows, Animals. (2021) 11, no. 1, 10.3390/ani11010165. PMC7828140 33445688

[17] Kaurivi Y. B. , Laven R. A. , Hickson R. , Parkinson T. P. , and Stafford K. J. , Assessing Extensive Cow-Calf Welfare in Namibia: Feasibility of Adapting a New Zealand Beef Cow Welfare Assessment Protocol, Journal of Applied Animal Welfare Science. (2023) 26, no. 1, 91–101, 10.1080/10888705.2021.1937168. 34541975

[18] Gobierno Departamental del Vichada , Plan Departamental de Desarrollo: Trabajo Para Todo Vichada 2020–2023, 2020, https://www.obsgestioneducativa.com/wp-content/uploads/2021/02/Vichada.pdf.

[19] Ramírez-Restrepo C. A. , Vera-Infanzón R. R. , and Rao I. M. , Predicting Methane Emissions, Animal-Environmental Metrics and Carbon Footprint From Brahman (*Bos indicus*) Breeding Herd Systems Based on Long-Term Research on Grazing of Neotropical Savanna and Brachiaria Decumbens Pastures, Agricultural Systems. (2020) 184, 10.1016/j.agsy.2020.102892.

[20] ICA , Estimaciones Poblacionales del Sector Pecuario, 2024, Censos Pecuarios Nacional, https://www.ica.gov.co/areas/pecuaria/servicios/epidemiologia-veterinaria/censos-2016/censo-2018.

[21] Landis J. R. and Koch G. G. , The Measurement of Observer Agreement for Categorical Data, Biometrics. (1977) 33, no. 1, 10.2307/2529310. 843571

[22] Ospina Rios S. L. , Lee C. , Andrewartha S. J. , and Verdon M. , A Pilot Study on the Feasibility of an Extended Suckling System for Pasture-Based Dairies, Animals. (2023) 13, no. 16, 10.3390/ani13162571. PMC10451218 37627361

[23] ICA , FNG , Agrosavia et al., Metodología Para la Evaluación de Bienestar Animal en Las Especies Bovina y Bufalina. Version 1, 2022, https://www.ica.gov.co/getattachment/Areas/Pecuaria/Servicios/Inocuidad-en-las-Cadenas-Agroalimentarias/Bienestar-Animal/Metodologia-bienestar-en-bovinos-y-bufalos.pdf.aspx?lang=es-CO#:%7E:text=Paralamedici%f3ndelbienestar,muestradeanimales%2Cyla.

[24] Rault J.-L. , Waiblinger S. , Boivin X. , and Hemsworth P. , The Power of a Positive Human–Animal Relationship for Animal Welfare, Frontiers in Veterinary Science. (2020) 7, 10.3389/fvets.2020.590867. PMC7680732 33240961

[25] Vigors B. and Lawrence A. , What are the Positives? Exploring Positive Welfare Indicators in a Qualitative Interview Study With Livestock Farmers, Animals. (2019) 9, 10.3390/ani9090694. PMC6770310 31533328

[26] Bjørkhaug H. , Sustainable Agriculture in the Norwegian Farmers’ Context: Exploring Farming Habitus and Practice on the Norwegian Agricultural Field, International Journal of Environmental, Cultural, Economic and Social Sustainability. (2006) 2, no. 4, 123–132, 10.18848/1832-2077/CGP/v02i04/54241.

[27] Logstein B. and Bjørkhaug H. , Good Animal Welfare in Norwegian Farmers’ Context. Can Both Industrial and Natural Conventions be Achieved in the Social License to Farm?, Journal of Rural Studies. (2023) 99, 107–120, 10.1016/j.jrurstud.2023.03.002.

[28] Bassi E. M. , Goddard E. , and Parkins J. R. , That’s the Way We’ve Always Done It: A Social Practice Analysis of Farm Animal Welfare in Alberta, Journal of Agricultural and Environmental Ethics. (2019) 32, no. 2, 335–354, 10.1007/s10806-019-09777-0.

[29] Falla-Tapias S. , Sierra-Barón W. , López-Santamaria E. , Botero-Aldana D. , and Burgos-Paz W. , Social Representations of Animal Health and Welfare in Rural Colombia: Implications for Sustainable Livestock Farming, Sustainability. (2025) 17, no. 11, 10.3390/su17115168.

[30] Peñuela L. , Solano C. , Ardila V. et al., Sabana Inundable y Ganadería, Opción Productiva de Conservación en la Orinoquia, Conservación de la Biodiversidad en Predios Productivos, Villavicencio. (2014) .

[31] Danieli B. , de Vitt M. G. , Schogor A. L. B. , Zotti M. L. A. N. , Ferraz P. F. P. , and Zampar A. , Effect of Grazing on the Welfare of Dairy Cows Raised Under Different Housing Conditions in Compost Barns, Animals. (2024) 14, no. 23, 10.3390/ani14233350. PMC11639460 39682315

[32] Phillips C. J. C. , Farm Animal Welfare—From the Farmers’ Perspective, Animals. (2024) 14, no. 5, 10.3390/ani14050671. PMC10931348 38473056

[33] Mee J. F. and Boyle L. A. , Assessing Whether Dairy Cow Welfare is “Better” in Pasture-Based Than in Confinement-Based Management Systems, New Zealand Veterinary Journal. (2020) 68, no. 3, 168–177, 10.1080/00480169.2020.1721034. 31973680

[34] Levallois P. , Buczinski S. , Desmarchelier M. , Lupien S. , and Robichaud M. V. , Relationships Between Farmer Well-Being and the Welfare of Their Animals: A One Welfare Scoping Review, Animal Welfare. (2026) 35, 10.1017/awf.2025.10056. PMC12817228 41567519

[35] Hampton J. O. , Jones B. , and McGreevy P. D. , Social License and Animal Welfare: Developments From the Past Decade in Australia, Animals. (2020) 10, no. 12, 10.3390/ani10122237. PMC7760378 33260531

[36] Mason W. A. , Laven L. J. , Huxley J. N. , and Laven R. , Can Lameness Prevalence in Dairy Herds be Predicted, From Farmers, Reports of Their Motivation to Control Lameness and Barriers to Doing So? An Observational Study From New Zealand, Journal of Dairy Science. (2024) 107, no. 4, 2332–2345, 10.3168/jds.2023-23862. 37863289

[37] Moreira T. F. , Nicolino R. R. , de Andrade L. S. , Filho E. J. F. , and de Carvalho A. U. , Prevalence of Lameness and Hoof Lesions in Grazing Cattle in Brazil, Tropical Animal Health and Production. (2018) 50, no. 8, 1829–1834, 10.1007/s11250-018-1626-3. 29846882

[38] Ranjbar S. , Rabiee A. R. , Gunn A. , and House J. , Identifying Risk Factors Associated With Lameness in Pasture-Based Dairy Herds, Journal of Dairy Science. (2016) 99, no. 9, 7495–7505, 10.3168/jds.2016-11142. 27394954

[39] Bran J. A. , Daros R. R. , von Keyserlingk M. A. G. , LeBlanc S. J. , and Hötzel M. J. , Cow- and Herd-Level Factors Associated With Lameness in Small-Scale Grazing Dairy Herds in Brazil, Preventive Veterinary Medicine. (2018) 151, 79–86, 10.1016/j.prevetmed.2018.01.006. 29496110

[40] Tsaousioti A. , Basioura A. , Praxitelous A. , and Tsousis G. , Dystocia in Dairy Cows and Heifers: A Review With a Focus on Future Perspectives, Dairy. (2024) 5, no. 4, 655–671, 10.3390/dairy5040049.

[41] Thrift F. A. and Thrift T. A. , Longevity Attributes of *Bos indicus* × *Bos taurus* Crossbred Cows, Professional Animal Scientist. (2003) 19, no. 5, 329–341, 10.15232/S1080-7446(15)31438-8.

[42] Kennedy E. , Murphy J. P. , Delaby L. , and O’Donovan M. , Effects of Short-Term Once-a-Day Milking in Early Lactation on Dairy Cow Performance When Managed in a Seasonal-Calving Pasture Based System, Livestock Science. (2021) 251, 10.1016/j.livsci.2021.104648.

[43] McDermott P. , McKevitt A. , Santos F. H. , and Hanlon A. , Management of Acutely Injured Cattle by on Farm Emergency Slaughter: Survey of Veterinarian Views, Frontiers in Veterinary Science. (2022) 9, 10.3389/fvets.2022.976595. PMC9686391 36439360

[44] McDermott P. , McKevitt A. , Santos F. H. , and Hanlon A. J. , Farmers Views on the Implementation of On-Farm Emergency Slaughter for the Management of Acutely Injured Cattle in Ireland, Animals. (2023) 13, no. 3, 10.3390/ani13030450. PMC9913314 36766340

[45] McLennan K. M. , Why Pain is Still a Welfare Issue for Farm Animals, and How Facial Expression Could be the Answer, Agriculture. (2018) 8, 10.3390/agriculture8080127.

[46] Roche S. , Saraceni J. , Zehr L. , and Renaud D. , Pain in Dairy Cattle: A Narrative Review of the Need for Pain Control, Industry Practices and Stakeholder Expectations, and Opportunities, Animals. (2025) 15, no. 6, 10.3390/ani15060877. PMC11939162 40150408

[47] Robles I. , Arruda A. G. , Nixon E. et al., Producer and Veterinarian Perspectives Towards Pain Management Practices in the US Cattle Industry, Animals. (2021) 11, no. 1, 10.3390/ani11010209. PMC7830793 33467105

[48] Vera-Infanzón R. R. , Ramírez-Restrepo C. A. , and Rao I. M. , Intensifying Neotropical Beef Cattle Grazing Systems: Navigating Complexity Through Modelling, Agricultural Systems. (2025) 226, 10.1016/j.agsy.2025.104301.

