# Supplementary material for: Animal Welfare Assessment in Cattle Breeding in the Natural Savannah of the Colombian Orinoquia
Source: Vet Med Int. 2026 Aug 12;2026:3163578. doi: 10.1155/vmi/3163578 (PMC13463238; doi:10.1155/vmi/3163578)
Supplement: Supplementary file 1 [file VMI-2026-3163578-s001.docx]

**Supplementary Material**

**APPENDIX 1**. Description of the indicators used in the animal welfare assessment protocol on cattle farms under grazing conditions in the natural savannah of the Colombian Orinoquia región.

| **AW principle** | **AW Criteria** | **AW measure/indicator** | **Description of indicator** | **Assessment method** | **Categorical classification** |
| --- | --- | --- | --- | --- | --- |
| **Good nutrition** | Absence of hunger | Access, quality, and quantity of food | Subjective assessment of the amount of grass in pastures (type and availability throughout the year) as sufficient or insufficient | Direct observation | Compliance with criteria  Low: 0 to 5/10  Medium: 6 to 8/10  High: 9 to 10/10 Excellent: 10/10 |
|  |  | Rumen filling * | Percentage of animals with empty rumen in summer and winter |  | ≤5  5.1-10%  >10% |
|  |  | Body condition | Percentage of thin animals in the herd, based on a score ≤ 2.5 on a scale of 1 to 5 Categorical scale based on the proportion of animals with a score ≤ 2.5 to assign a total score (0 to 8). | Animal-based indicator (overall average per farm) | Categorical scale  High: 3–3.5  Excellent: 4–5 |
|  | Absence of thirst | Access, availability, quality, and quantity of water in drinking troughs and natural sources | Subjective assessment of the availability of natural water sources/drinking fountains as sufficient or insufficient | Interview and direct observation | Compliance with criteria  Low: 0 to 5/10  Medium: 6 to 8/10  High: 9/10  Excellent: 10/10 |
|  |  | Distance to water access* | Average distance to water |  | 0-250 m  250-500 m  >500 m |
| **Appropriate environment** | Thermal comfort | Adaptation of animals to the temperature and humidity conditions of the area | Subjective assessment of  animals' adaptation to the climate | Interview and direct observation | Criteria compliance  Low: 0 to 6/12  Medium: 7 to 8/12  High: 9 to 11/12 Excellent: 12/12 |
|  |  | Protection from weather effects | Subjective assessment of shade in pastures (presence of shrubs, trees, and galleries) |  |  |
|  |  | Adaptations that provide greater comfort to the animal | Scratching posts, trees, or forest that allow them to explore or hide and have options other than grass for  foraging |  |  |
|  | Comfort around the resting area | Clean and dry resting area | Subjective assessment of potential resting places |  |  |
|  |  | General condition of the pastures |  |  |  |
|  |  | Overall condition of facilities |  |  |  |
|  |  | Presence of hazards* | Identification of hazards observed in pastures (physical: animals stuck in mud, predators: felines  ophidians: snakes | Interview and direct observation | 3-point categorical scale.  1: No hazards  2: 1-2 hazards  3: >3 dangers |
| **Good health** | Absence of pain due to handling procedures | Identification/Tagging/Castration | Age of animal, staff experience, and use of analgesics and local anesthetics | Interview, records, and direct observation | Compliance with criteria  Low: 0 to 4/8  Medium: 5 to 6/8  High: 7/8  Excellent 8/8 |
|  | Absence of disease and pain | Absence of abrasions/inflammation/alopecia | Percentage of animals with recent injuries | Indicators based on animals  (overall average per farm) | Animals presenting  Low: 90–100% (0/3)  Medium: ≥ 70% (1/3)  High: 50-69% (2/3)  Excellent <50% (3/3) |
|  |  | Presence of ectoparasites | % of animals with ectoparasites (ticks, flies, lice, and myiasis) |  |  |
|  |  | Lameness | On pasture/subjective assessment Percentage of cattle with uneven weight bearing on one limb that is immediately identifiable and/or clearly shortened stride. | Direct observation  (overall average per farm) | Animals without lameness  Low: <50% (0/3)  Medium: 50-89% (1/3)  High: ≥ 90% (2/3)  Excellent 100% (3/3) |
|  |  | Animals requiring additional care | % of sick or injured animals that do not receive timely treatment and care (separation from the herd, provision of soft bedding, access to water and food, treatment) | Animal-based indicator (overall average per farm) | Animals requiring additional care  Low: >5% (0/3)  Medium: 2.1–5% (1/3)  High: 1.1–2% (2/3)  Excellent ≤ 1% (3/3) |
|  |  | Mortality rate | % of animals killed in accidents and deaths/slaughter (on the farm or sent off the farm) due to disease in the 12 months prior to the assessment | Calculation, interview, and records | Mortality  Low: >5% (0/3)  Medium: 3.1-5% (1/3)  High: 2.1-3% (2/3)  Excellent < 2% (3/3) |
|  |  | Culling rate | Percentage of animals that have been discarded in the last 12 months | Interview and records | Excellent: ≤20% of animals  Low: > 20%  Compliance with criteria  Low: 1/6 or <  Medium: 2/6  High: 3 to 4/6  Excellent: 5 to 6/6 |
|  |  | Health checks * | Frequency of animal health checks |  | Checks  Low: more than 3 weeks  Medium: 1–2 weeks  Excellent: every week |
|  |  | Mastitis | Percentage of animals with mastitis in the last 12 months |  | Mastitis  Low: >3% (0/3)  Medium: 2.1-3% (1/3)  High: 1.1-2% (2/3)  Excellent: ≤1% (3/3) |
|  |  | Dystocia * | % of cows with dystocia |  | Dystocia  Low: >2%  Medium: 0.1-2%  Excellent: 0% |
| **Appropriate behavior** | Human-animal interaction | Animal reaction to humans  (Calm/excitable) | Assessment of animal behavior when the rider enters the pasture (animal reaction and movement) Categories: Calm-calm (animals remain still, with little or no resistance when approached)/ Excitable (constant, vigorous movement, attempting to escape, very agitated and frightened) | Animal-based indicators  (overall average per farm) | Compliance with criteria  Low: 0-2  Medium: 3-4  High: 5/6  Excellent: 6/6 |
|  |  | Human-animal interaction | Assessment of the animal's orientation response towards the handler. Categories: Positive: relaxed head, ears, and body posture, showing interest in the handler. Negative: vigilance, avoidance, flight. Neutral: head in normal position and ears erect while looking at the handler, continuous rumination. |  |  |
|  |  | Knowledge and training in animal welfare | Percentage of people with certified training in animal welfare | Interview, certificates | Low: n/a  Medium: <50%  High: ≥50%  Excellent: 100% |
|  |  | Dairy farming method* | Method used for dairy farming activity | Interview and direct observation | (on foot/horseback/motorcycle/other) |
|  |  |  |  |  |  |

Table modified from ICA et al. [14]; M. H. Romero et al. [3] and Kaurivi et al.[13] *.
